# Effectiveness of Continuum of Care—Linking Pre-Pregnancy Care and Pregnancy Care to Improve Neonatal and Perinatal Mortality: A Systematic Review and Meta-Analysis

**DOI:** 10.1371/journal.pone.0164965

**Published:** 2016-10-27

**Authors:** Kimiyo Kikuchi, Sumiyo Okawa, Collins O. F. Zamawe, Akira Shibanuma, Keiko Nanishi, Azusa Iwamoto, Yu Mon Saw, Masamine Jimba

**Affiliations:** 1 Department of Community and Global Health, Graduate School of Medicine, the University of Tokyo, Tokyo, Japan; 2 Institute of Decision Science for a Sustainable Society, Kyushu University, Fukuoka, Japan; 3 Office of International Academic Affairs, Graduate School of Medicine, the University of Tokyo, Tokyo, Japan; 4 Bureau of International Medical Cooperation, National Center for Global Health and Medicine, Tokyo, Japan; 5 Department of Healthcare Administration, Graduate School of Medicine, Nagoya University, Nagoya, Japan; 6 Nagoya University Asian Satellite Campuses Institute, Nagoya, Japan; Centre Hospitalier Universitaire Vaudois, FRANCE

## Abstract

In an era of Sustainable Development Goals, maternal, newborn, and child health still require improvement. Continuum of care is considered key to improving the health status of these populations. The continuum of care is a series of care strategies starting from pre-pregnancy to motherhood-childhood. The effectiveness of such linkage between the pregnancy, birth, and postnatal periods has been demonstrated. However, almost no study has assessed the impact of linkage that starts from pre-pregnancy to pregnancy care on maternal and child health. The present study attempts to fill this gap by assessing the effectiveness of the care linkage between pre-pregnancy and pregnancy care for reducing neonatal, perinatal, and maternal mortality in low- and middle-income countries. We performed a systematic review and meta-analysis of randomized and quasi-randomized controlled trials in low- and middle-income countries. The outcome variables were neonatal, perinatal, and maternal mortality. We searched databases such as PubMed/Medline, POPLINE, EBSCO/CINAHL, and ISI Web of Science for the period 2000–2014, using broad search terms (e.g., pre-pregnancy OR adolescent OR mother), combined with search terms specific for interventions, (e.g., family planning OR contraception OR spacing). From the 1,325 retrieved articles, five studies were finally analyzed. The meta-analysis showed that interventions linking pre-pregnancy and pregnancy care effectively reduced neonatal mortality (risk ratio [RR]: 0.79; 95% confidence interval [CI]: 0.71–0.89, I^2^ = 62%) and perinatal mortality (RR: 0.84; 95% CI: 0.75–0.94, I^2^ = 73%), but did not show an effect on maternal mortality. Neonatal and perinatal mortality could be reduced by linking pre-pregnancy and pregnancy care. This linkage of pre-pregnancy and pregnancy cares is an essential component of continuum of care to improve newborn health.

**Review Registration:**

PROSPERO International prospective register of systematic reviews (CRD42015023424).

## Introduction

The United Nations’ Millennium Development Goals called on countries to improve maternal, newborn, and child health. Since 1990, the maternal mortality ratio has declined by 45% and the under-five mortality rate has fallen from 90 to 43 deaths per 1,000 live births [1]. However, inequality of health service coverage remains high in low- and middle-income countries [2]; more lives could be saved by improving their access to care. Thus, in an era of Sustainable Development Goals, maternal, newborn, and child health still require improvement [3].

The concept of continuum of care has been advocated as a means of improving maternal, newborn, and child health [4]. Continuum of care is well known in clinical medicine in HIV care and nursing care [5]. While it is defined as a continuous care of non-curable conditions, continuum of care in maternal, newborn, and child health is a series of necessary care strategies for women and children to avoid preventable diseases. It has two dimensions: a time dimension from pre-pregnancy through pregnancy, birth, the postnatal period, through to childhood, and a space dimension from community-family care to clinical care [6]. A meta-analysis has shown the effectiveness of interventions linking antenatal to postnatal care in improving neonatal and perinatal deaths [7]. However, most of the previous continuum of care studies have mainly focused on the pregnancy, birth, and postnatal periods. Almost no attempts have been made to assess how maternal and newborn health can be improved by interventions linking care from pre-pregnancy to pregnancy periods.

Pre-pregnant women, including adolescents, are a critical population group. They are at the beginning of the continuum of care in maternal, newborn, and child health and events during pre-pregnancy could affect their health throughout the lifespan [8]. Adolescent women are generally healthy, but need help to acquire the right to develop their full potential as mothers [9]. Consequently, various health services and interventions have been developed for them. For pre-pregnant women, nutrition care and healthy behavior need to be promoted to enhance maternal physical development [10]. For example, anemia is a very common nutritional problem among women and the risk of maternal mortality for severe anemia is 3.51 [11]. To avoid adverse pregnancy outcomes, pre-pregnant women need to think of their sexual health and prevent or manage sexually transmitted illnesses (STIs) [8]. Syphilis results in a 15% risk of stillbirth, a 14% risk of neonatal death, and only a 20% chance of giving birth to a healthy, uninfected infant [12]. Pre-pregnant women must also try to prevent complication risks, such as obesity and diabetes [13]. Risk of a cesarean delivery were 2.89 times higher among severely obese women [14]. Above all, family planning should be promoted regardless of a woman’s willingness to conceive [15], as interpregnancy intervals shorter than 6 months increase the risk of preterm birth by 1.40 times and low birth weight by 1.61 times [16]. Early childbearing women, under 20 years of age, face a 50% higher risk of having a stillbirth or of the baby dying in the first few weeks compared to mothers aged 20–29 [17]. Consensus has already been reached among health care providers that pre-pregnancy care can increase the health and well-being of women and child [15, 18].

Although the continuum of care linking pre-pregnancy and pregnancy is expected to be highly effective, no prior systematic review article or protocol is available in this area. If the effectiveness of the linkage is analyzed, it could reveal further opportunities for intervening in maternal, newborn, and child health care and offer insights for tailoring interventions. Thus, via a systematic review and meta-analysis, we quantitatively synthesized evidence of the effectiveness of the continuum of care between pre-pregnancy and pregnancy care in low- and middle- income countries. Furthermore, we examine its impact on maternal and newborn health outcomes.

## Methods

We conducted a systematic review and meta-analysis following the guidelines of the Cochrane Collaboration [19]. It follows the four phases indicated in the Preferred Reporting Items for Systematic Reviews and Meta-Analyses (PRISMA) Statement [20]. Our systematic review was developed based on the PRISMA checklist as presented in S1 File. We registered the protocol for this systematic review at the PROSPERO International prospective register of systematic reviews on June 12, 2015. We then updated it on September 15, 2015 (registration number: CRD42015023424; available at http://www.crd.york.ac.uk/PROSPERO).

### Study inclusion criteria

We included only randomized and quasi-randomized controlled trials in this systematic review and meta-analysis, including both individual and cluster-randomized studies. We selected only peer-reviewed journals and reports from international organizations as publication types. We considered papers in all languages as long as they had English abstracts. Furthermore, we included only studies located in low- and middle-income countries as defined by the World Bank [21]. We excluded non-randomized studies and non-intervention studies such as case series, case reports, and qualitative studies, and studies conducted in high-income countries.

### Participants

We included pre-pregnant women of reproductive age and maternal and child healthcare service providers residing in low- and middle-income countries as participants. Women of reproductive age were defined as 15–49 years old following the World Health Organization’s (WHO’s) definition. We excluded studies that included specific subpopulations that would complicate generalizing the results to a wider population, such as HIV-positive women and groups at high risk for pregnancy complications.

### Interventions and controls

Interventions comprised packaged care/services that addressed women’s time dimension from pre-pregnancy to pregnancy. The space dimension of continuum of care comprises three care stages—community/family care, outpatient/outreach care, and clinic care [22]. The community/family care interventions included home or community-based care practices addressing pre-pregnant women’s nutrition, health education for family planning and reproductive health, and prevention of HIV/STIs. The outpatient/outreach care included family planning and prevention/management of HIV/STIs. The clinical care interventions included elective abortion and post-abortion care through facility-based care at primary and referral levels.

We defined the control groups as those who received standard care. In this study, standard care was defined as that provided in health facilities according to local or national guidelines.

### Outcomes

We included studies assessing any of the outcomes below.

1. Neonatal mortality
2. Maternal mortality
3. Perinatal mortality

Since mortality is often the main outcome measure in maternal, newborn, and child health studies, we selected several types as outcomes [23]. We also included studies containing data from which we could calculate these outcomes.

### Search strategy

We searched articles in the following bibliographic databases: PubMed/Medline, POPLINE, EBSCO/CINAHL, BiblioMap, and ISI Web of Science. We also reviewed relevant internet sources from the WHO library database and Google Scholar for additional grey literature. Finally, we searched for additional studies using the snowball method of reviewing the reference lists of retrieved articles. We limited the publication period to 15 years, from 2000 to 2014, to ensure that we retrieved a sufficient number of studies.

We prepared an inventory of the intervention aims to reduce maternal and child morbidity or unmet pregnancy needs using the key interventions recommended by the WHO [24]. As for pre-pregnancy care, the interventions were healthcare service provision or health education relevant to family planning, STIs, and nutrition provided in health facilities or communities. Regarding pregnancy care, the interventions were the provision of appropriate antenatal care by healthcare providers including screening for maternal illnesses, defining possible complications, tetanus immunization, birth preparedness, malaria prevention, and smoking cessation. For literature searches, we used appropriate key words, accepted MeSH words, and combinations thereof. One search approach employed broad search terms (e.g., “pre-pregnancy” [MeSH] OR adolescent OR mother), combined with search terms specific for interventions, (e.g., “family planning” [MeSH] OR contraception OR spacing). The specific electronic search strategy is provided in S2 File.

### Data collection and analysis

#### Selection of studies

**Fig 1:**
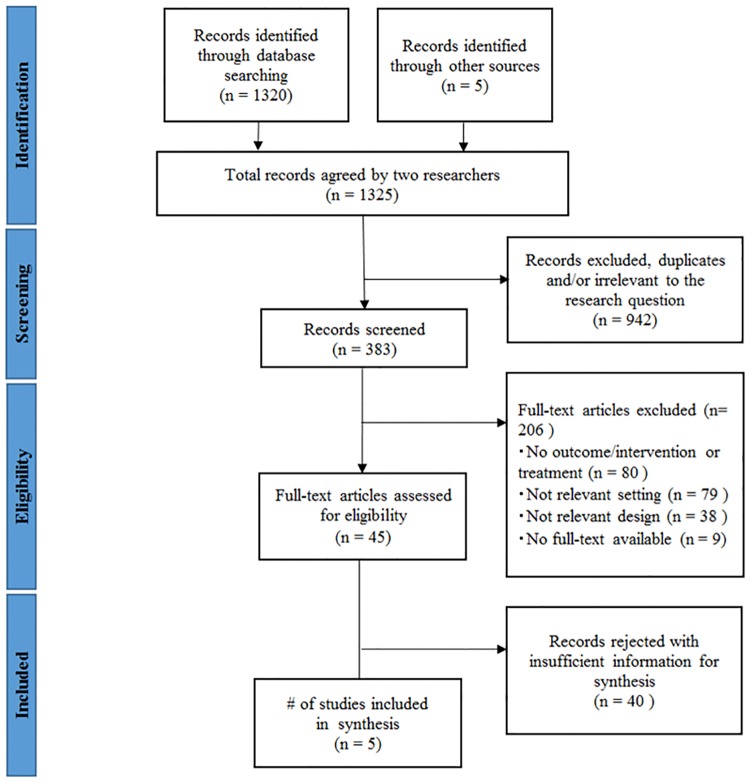
Diagram of information flow through phases of systematic review.

The study selection process is summarized in the flowchart in Fig 1. Three authors (KK, SO, and CZ) independently extracted data and screened the quality and content of the included studies. We identified articles by analyzing the titles and abstracts for relevance and compliance with the selection criteria based on the research setting, study design, and reported outcomes. We classified articles as included, excluded, uncertain, or duplicate. We confirmed all potential included or uncertain studies and resolved any disagreements by consensus.

#### Data extraction and management

KK and SO extracted the features of each study (e.g., study design, setting, components of intervention package, and outcomes) and entered them into a standardized form. KK extracted data and SO checked them for accuracy and completeness. Again, when they noted discrepancies, the two authors discussed until they reached an agreement.

#### Assessment of Risk of Bias on included studies

KK and SO assessed the quality of trials using the “risk of bias” tool presented in the Cochrane Handbook for Systematic Reviews of Interventions [19]. Specifically, we assessed the risk of bias in seven domains: sequence generation, allocation concealment, blinding of participants and outcome assessors, incomplete outcome data, selective outcome reporting, and other potential threats to validity. We resolved all discrepancies by consensus.

Information regarding the risk of bias is shown in Table 1. Two studies lacked information related to the risk of bias or these risks were unclear. Due to the nature of the study design, allocation concealment was not an issue in the cluster-randomized studies we extracted [19]. However, we counted baseline imbalance in determining selection bias.

**Table 1:** Risk of bias for randomized and quasi-randomized studies.

	Azad 2010	Bhutta 2008	Bhutta 2011	Manandhar 2004	Tripathy 2010
Random sequence generation (selection bias)	+	?	+	+	+
Allocation concealment (selection bias)	+	+	+	+	+
Blinding of participants and personnel (performance bias)	-	?	?	-	-
Blinding of outcome assessment (detection bias)	-	-	+	+	+
Incomplete outcome data (attrition bias)	+	?	?	+	+
Selective reporting (reporting bias)	+	+	+	+	+
Other bias	+	-	+	+	-

+: low risk of bias, -: high risk of bias,?: unclear

#### Measures of treatment effect

We presented results as risk ratios (RR) with 95% confidence intervals (CI) for all randomized and quasi-randomized controlled trials. We checked whether the studies contained a unit of analysis error. In cases where we observed this error, we re-analyzed the available data. However, we did not find obvious analysis errors in the identified articles.

#### Analysis

First, we narratively summarized the included study interventions. Second, we stratified the studies by service delivery mode with the RRs of applied mortality. Third, we conducted a meta-analysis for mortality, using the software Review Manager (RevMan). Version 5.1. (Copenhagen, the Nordic Cochrane Centre, the Cochrane Collaboration). We assessed the heterogeneity with the I^2^ statistic and a significance threshold of 0.10. We used random-effects models to adjust for possible heterogeneity. Publication bias was assessed using a funnel plot.

## Results

We retrieved 1,325 articles from the sources (Fig 1). Of these, 502 studies were identified from PubMed, 474 were from POPLINE, 207 were from EBSCO/CINAHL, 137 were identified through other databases, and five by through hand searching. After an initial screening, we included 383 studies because others were duplicates or irrelevant to the research question. Of the remaining studies, we excluded a further 206 because they had no outcome/intervention or treatment (n = 80), irrelevant setting (n = 79), irrelevant design (n = 38), or no full-text available (n = 9; multiple reasons were possible). We assessed the remaining 45 studies for eligibility through review of the full-text articles. After this full-text review, we included five studies in the analysis [25–29]. They were all written in English and had been published in peer-reviewed journals. As indicated in Table 2, all employed cluster-randomized controlled trial designs and were conducted in Asia. All studies contained community-based activities and linked time dimensions of continuum of care between pre-pregnancy care and pregnancy care. Three studies contained interventions expanding community/family care to clinical care [27–29]. The other two studies involved only community/family care interventions [25, 26].

**Table 2:** Characteristics of studies that intervened pre-pregnancy and pregnancy care.

Time dimension	Space dimension	Author/Country	Study design	Participants	Intervention	Outcome	Number of participants
Pre-pregnancy Pregnancy Birth Postnatal	Community-familycare Outpatient-outreach care Clinical care	Azad et al. 2010, Bangladesh	Cluster randomized controlled trial	Women aged 15–49 years	Maternal and neonatal health promotion for reproductive age women provided by women's groups in the community/Training for traditional birth attendants on safe deliveries and resuscitation of newborns with symptoms of birth asphyxia using the bag valve mask /Basic and refresher clinical training for health workers on essential components of maternal and neonatal health care.	Neonatal death Maternal death Stillbirth	Intervention arm: 15,695 births Control arm: 15,257 births (twins were included and temporary residents were excluded)
Pre-pregnancy Pregnancy Postnatal	Community-family care Outpatient-outreach care Clinical care	Bhutta et al. 2008, Pakistan	Cluster randomized controlled trial	Women of reproductive age, adolescent girls, and older women	Training for female health workers to provide home visits to pregnant and postpartum women/Set up community health committees to conduct 3-monthly group education sessions in villages for women of reproductive age, adolescent girls, and older women/Established an emergency transport fund for mothers and newborns/Education on basic and intermediate newborn care for health workers/Specialized training for medical and nursing staff.	Neonatal death Stillbirth Perinatal death	Intervention arm: 3,064 births Control arm: 2,778 births Intervention arm: 395 pregnancies Control arm: 375 pregnancies
Pre-pregnancy Pregnancy Birth Postnatal	Community-family care Outpatient-outreach care Clinical care	Bhutta et al. 2011, Pakistan	Cluster randomized controlled trial	Women of reproductive age, adolescent girls, and older women	Group health education sessions for women on antenatal care and maternal health by female health workers/Provision of clean delivery kits/Promotion of health facility delivery, immediate newborn care, and training in the identification of danger signs/Instruction on antenatal and postnatal home visits for female health workers/Training for traditional birth attendants on basic newborn care/Establishing community health committees for maternal and newborn care.	Neonatal death Stillbirths Perinatal death	Intervention arm: 12,028 births Control arm: 11,005 births Intervention arm: 2,339 deliveries Control arm: 2,135 deliveries
Pre-pregnancy Pregnancy Birth Postnatal	Community-family care	Manandhar et al. 2004, Nepal	Cluster randomized controlled trial	Women aged 15–49 years with the potential to become pregnant	Monthly women's group meetings with female facilitators/Activate a women's group through an action-learning cycle/Assisted the women's group in identifying and prioritizing maternal and neonatal problems/Helping the women's group to identify possible solutions and to plan, implement and monitor the solution strategies in the community/Essential newborn care training for government health staff, female community health volunteers and traditional birth attendants.	Neonatal death Stillbirth Maternal death	Intervention arm: 2,972 infants born/3190 pregnancies Control arm: 3,303 infants born/ 3524 pregnancies (twins were included)
Pre-pregnancy Pregnancy Birth Postnatal	Community-family care	Tripathy et al. 2010, India	Cluster randomized controlled trial	Women aged 15–49 years who became pregnant during the study period	Training for facilitators who activate women's groups/Assisted women's groups to identify and prioritize maternal and neonatal problems/Helped women's groups to identify possible solutions and to plan, implement and monitor solution strategies in the community.	Neonatal death Stillbirth Maternal death Perinatal death	Intervention arm: 9,770 births Control arm: 9,260 births (migrated mothers and infants were excluded)

All identified studies did not provide specific pre-pregnancy care, but intervened with pre-pregnant women as a part of the target group. The main components of intervention were general maternal health education or its combination with family planning or sexual health. Three studies examined interventions for women in the community that employed participatory approaches by women’s groups [25–27]. Groups were trained specifically to promote maternal and child health. Group members provided collective education sessions to reproductive aged women. Two studies were interventions developed mainly by female health workers who had a mission to provide home-based health education and maternal and childcare [28, 29]. All studies estimated neonatal and perinatal mortality. Only three studies estimated maternal mortality [25–27].

### Impact of care linkage on health outcomes

**Fig 2:**
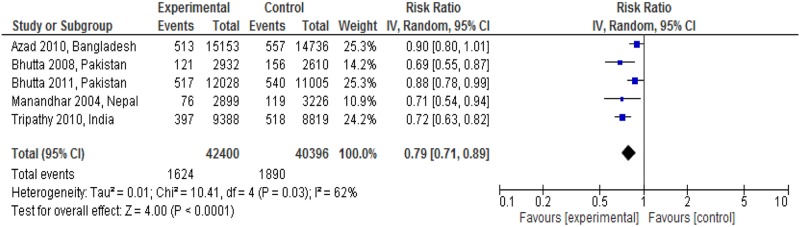
Neonatal mortality risk ratio for interventions linking pre-pregnancy and pregnancy care.

**Fig 3:**
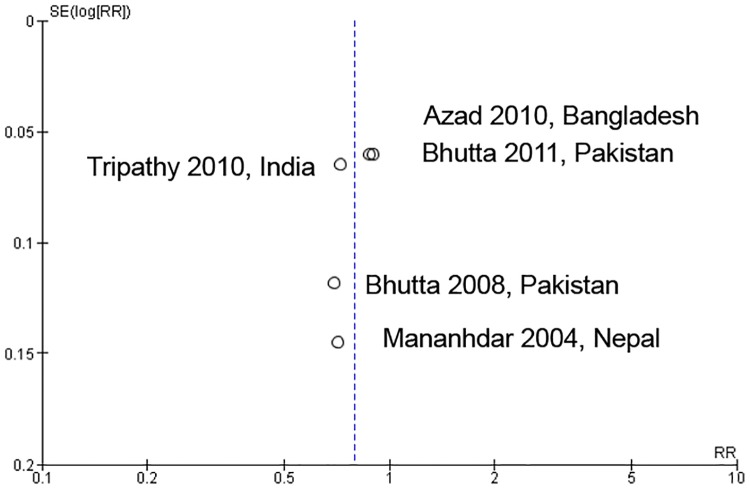
Funnel plot of interventions that assessed neonatal mortality risk ratio.

Fig 2 showed the neonatal mortality impact of linkages between pre-pregnancy care and pregnancy care. Four studies identified a significant reduction in neonatal mortality [10, 25, 26, 29]. The meta-analysis also showed a significant reduction in neonatal mortality (RR: 0.79; 95% CI: 0.71 to 0.89; random effects [five studies, n = 82,796], I^2^ = 62%). We did not find any obvious asymmetry in the funnel plot (Fig 3).

**Fig 4:**
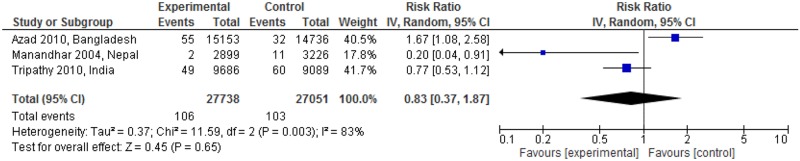
Maternal mortality risk ratio for interventions linking pre-pregnancy and pregnancy care.

**Fig 5:**
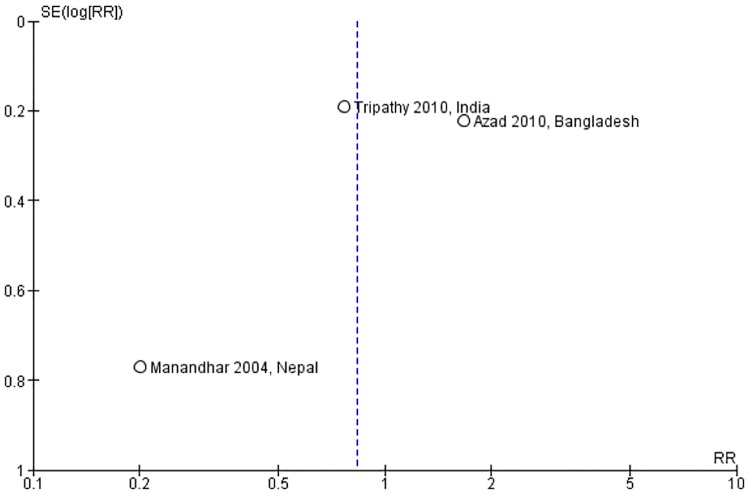
Funnel plot of interventions that assessed maternal mortality risk ratio.

Fig 4 showed the maternal mortality impact of linkages between pre-pregnancy care and pregnancy care. The linked intervention significantly reduced maternal mortality in one study (RR: 0.20; 95% CI: 0.04 to 0.91) [26], but increased it in another (RR: 1.67; 95% CI: 1.08 to 2.58) [27]. The meta-analysis did not show significant change in maternal mortality (RR: 0.83; 95% CI: 0.37 to 1.87; random effects [three studies, n = 54,789], I^2^ = 83%). The funnel plot appeared asymmetric among the studies (Fig 5).

**Fig 6:**
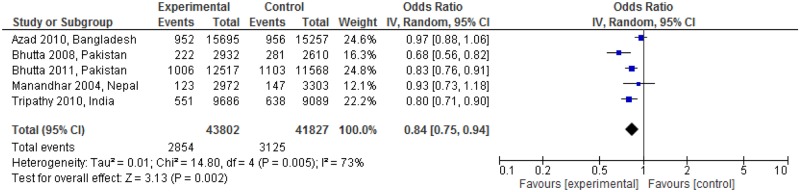
Perinatal mortality risk ratio for interventions linking pre-pregnancy and pregnancy care.

**Fig 7:**
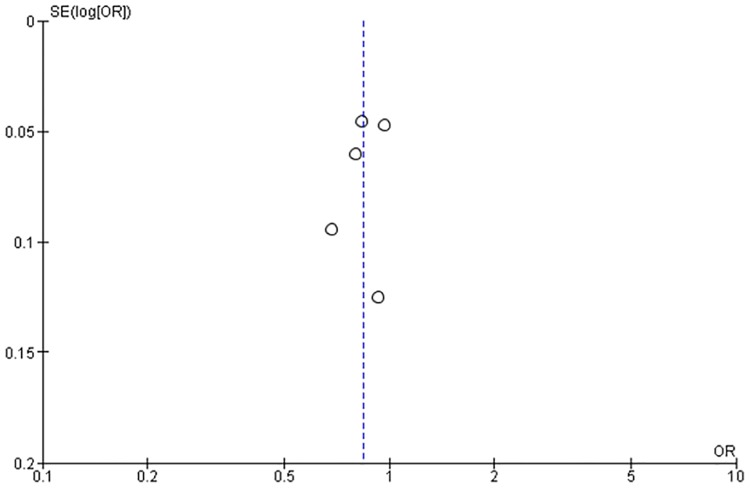
Funnel plot of interventions that assessed perinatal mortality risk ratio.

Fig 6 shows the impact on perinatal mortality regarding linkages between pre-pregnancy and pregnancy care. The linked intervention significantly reduced neonatal death in three studies (RR: 0.68; 95% CI: 0.56 to 0.82; RR: 0.83; 95% CI: 0.76 to 0.91; RR: 0.80; 95% CI: 0.71 to 0.90) [25, 28, 29]. There was a significant reduction in perinatal mortality rate (RR: 0.84; 95% CI: 0.75 to 0.94, random effects [five studies, n = 85,629], I^2^ = 73%). We did not find obvious asymmetry in the funnel plot (Fig 7).

## Discussion

This is the first systematic review to examine the effectiveness of interventions that linked pre-pregnancy care and pregnancy care in reducing maternal, neonatal, and perinatal mortality. In total, five studies were identified as eligible. Some new and important results were found. First, interventions linking pre-pregnancy and pregnancy care were effective for reducing neonatal and perinatal mortality. Second, they were not significantly effective in reducing maternal mortality.

Although continuum of care has been recommended for improving maternal and newborn health, only five studies were identified that demonstrated the effectiveness in linkages between pre-pregnancy and pregnancy care. Many of the studies were cross-sectional and did not conduct follow-ups to obtain longitudinal evidence on mortality [22]. This could be because the follow-up period is usually unpredictable for pre-pregnant women becoming pregnant. However, more concrete results are needed to understand the linkage of care. Thus, further research should be conducted on this topic in low- and middle-income countries.

In the studies that we identified, the pre-pregnancy care provided was mainly general maternal health education, whereas the WHO recommends other different health care services: family planning, prevention and management of STIs, and folic acid fortification/supplementation [24]. Furthermore, as the studies did not target only pre-pregnant women, it is difficult to ascertain to what extent the interventions influenced their health. Therefore, we are only able to describe rough patterns of the effectiveness of such continuum of care.

Nevertheless, although the interventions were limited mainly to health promotion, neonatal and perinatal mortality could be significantly reduced in interventions linking pre-pregnancy care and pregnancy care. From the detected studies, it was unclear whether pre-pregnancy care had a positive impact on pregnancy care. However, it is undeniable that pre-pregnancy health promotion led women to take more appropriate care [15, 30] and to seek early care as Tripathy stated [25]. Consequently, early breastfeeding practice and delays in bathing were significantly improved in the identified studies. In addition, neonatal and perinatal mortality improvement were attributed to those changes. The effectiveness of pre-pregnancy health promotion in birth outcome and mother’s health behavior was also reported in studies conducted in the USA and Australia [31–34].

In contrast to the above positive neonatal and perinatal health outcomes, a significant reduction for maternal mortality was not found. Effectiveness of intervention to maternal mortality was remarkably different from one study to another, thus the inconsistency level was elevated. This might have caused inconsistency between the studies. This inconsistency could be attributed to different reasons, but the quality of the intervention might have affected the maternal mortality as stated in Azad’s study [27]. It could be mitigated if the number of studies increases, thus further studies are needed for more accurate meta-analysis results. Another possible explanation for the insignificant result is that participants who received antenatal care more than three times were only 13%–41% in the studies included in our analysis. This suggests that most of the participants did not receive necessary and timely care while pregnant, and missed important treatment opportunities. Additionally, causes of stillbirths included congenital lesions or unexpected infectious diseases; health education has only a limited role in preventing such problems [35].

### Limitations

This study has two limitations. First, the lack of randomized studies may have obscured the real results of the meta-analysis. Further evidence is needed on the effectiveness of the continuum of care between pre-pregnancy and pregnancy periods.

Second, the studies we identified included interventions in the delivery and postpartum periods. Their results could be affected by those interventions. Thus, the present findings need to be interpreted with caution.

## Conclusions

Newborn health outcomes could be improved in low- and middle-income countries by mothers’ receiving continuous pre-pregnancy and pregnancy care. In particular, this continuum of care conclusively affected neonatal and perinatal mortality, but showed no evidence of an impact on maternal mortality in our meta-analysis. However, as primary evidence is scarce, further research is needed on continuum of care between pre-pregnancy and pregnancy to consolidate its effectiveness.

## Supporting Information

S1 File PRISMA 2009 Checklist. (DOC)

S2 File Systematic review protocol registration (PROSPERO). (PDF)

S3 File Search terms summary. (DOCX)
