# Supplementary material for: Effectiveness of Continuum of Care—Linking Pre-Pregnancy Care and Pregnancy Care to Improve Neonatal and Perinatal Mortality: A Systematic Review and Meta-Analysis
Source: PLoS One. 2016 Oct 27;11(10):e0164965. doi: 10.1371/journal.pone.0164965 (PMC5082954; doi:10.1371/journal.pone.0164965)
Supplement: S2 File — (PDF) [file pone.0164965.s002.pdf]

PROSPERO International prospective register of systematic reviews

Review title and timescale

1 Review title
Give the working title of the review. This must be in English. Ideally it should state succinctly the interventions or exposures being reviewed and the associated health or social problem being addressed in the review.
The effectiveness of integrating pre-pregnancy care and pregnancy care: a systematic review

2 Original language title
For reviews in languages other than English, this field should be used to enter the title in the language of the review. This will be displayed together with the English language title.

3 Anticipated or actual start date
Give the date when the systematic review commenced, or is expected to commence.
25/06/2015

4 Anticipated completion date
Give the date by which the review is expected to be completed.
25/08/2015

5 Stage of review at time of this submission
Indicate the stage of progress of the review by ticking the relevant boxes. Reviews that have progressed beyond the point of completing data extraction at the time of initial registration are not eligible for inclusion in PROSPERO. This field should be updated when any amendments are made to a published record.

The review has not yet started √

Review stage	Started	Completed
Preliminary searches	No	No
Piloting of the study selection process	No	No
Formal screening of search results against eligibility criteria	No	No
Data extraction	No	No
Risk of bias (quality) assessment	No	No
Data analysis	No	No

Provide any other relevant information about the stage of the review here.

Review team details

6 Named contact
The named contact acts as the guarantor for the accuracy of the information presented in the register record.
Masamine Jimba

7 Named contact email
Enter the electronic mail address of the named contact.
mjimba@m.u-tokyo.ac.jp

8 Named contact address
Enter the full postal address for the named contact.
7-3-1, Hongo, Bunkyo-ku, Tokyo, 113-0033

9 Named contact phone number
Enter the telephone number for the named contact, including international dialing code.
+81-3-5841-3698

10 Organisational affiliation of the review
Full title of the organisational affiliations for this review, and website address if available. This field may be completed as 'None' if the review is not affiliated to any organisation.
Department of Community and Global Health, Graduate School of Medicine, The University of Tokyo
Website address:
http://www.ich.m.u-tokyo.ac.jp/

11 Review team members and their organisational affiliations
Give the title, first name and last name of all members of the team working directly on the review. Give the organisational affiliations of each member of the review team.

Title	First name	Last name	Affiliation
Dr	Kimiyo	Kikuchi	The University of Tokyo
Dr	Junko	Yasuoka	The University of Tokyo
Dr	Keiko	Nanishi	The University of Tokyo
Mr	Akira	Shibanuma	The University of Tokyo
Dr	Sumiyo	Okawa	The University of Tokyo
Dr	Azusa	Iwamoto	National Center for Global Health and Medicine
Dr	Yumon	Saw	Nagoya University
Professor	Masamine	Jimba	The University of Tokyo

Dr Junko Yasuoka The University of Tokyo
Dr Keiko Nanishi The University of Tokyo
Mr Akira Shibanuma The University of Tokyo
Dr Sumiyo Okawa The University of Tokyo
Dr Azusa Iwamoto National Center for Global Health and Medicine
Dr Yumon Saw Nagoya University
ProfessorMasamine Jimba The University of Tokyo

12 Funding sources/sponsors
Give details of the individuals, organizations, groups or other legal entities who take responsibility for initiating, managing, sponsoring and/or financing the review. Any unique identification numbers assigned to the review by the individuals or bodies listed should be included.
The University of Tokyo Japan Agency for Medical Research and Development

13 Conflicts of interest
List any conditions that could lead to actual or perceived undue influence on judgements concerning the main topic investigated in the review.
Are there any actual or potential conflicts of interest?
None known

14 Collaborators
Give the name, affiliation and role of any individuals or organisations who are working on the review but who are not listed as review team members.

Title	First name	Last name	Organisation details

Review methods

15 Review question(s)
State the question(s) to be addressed / review objectives. Please complete a separate box for each question.
What is the impact on maternal health outcomes of integrating care from pre-pregnancy to pregnancy in low- and middle-income countries?
What is the impact on health service usage of integrating care from pre-pregnancy to pregnancy in low- and middle-income countries?

16 Searches
Give details of the sources to be searched, and any restrictions (e.g. language or publication period). The full search strategy is not required, but may be supplied as a link or attachment.
We will conduct literature search from different medical databases including : PubMed/MEDLINE, CINAHL, and ISI Web of Knowledge. The search is limited to 15 years of publication (2000 to 2015).

17 URL to search strategy
If you have one, give the link to your search strategy here. Alternatively you can e-mail this to PROSPERO and we will store and link to it.

I give permission for this file to be made publicly available
Yes

18 Condition or domain being studied
Give a short description of the disease, condition or healthcare domain being studied. This could include health and wellbeing outcomes.
Integration of health care from pre-pregnancy to pregnancy

19 Participants/population
Give summary criteria for the participants or populations being studied by the review. The preferred format includes details of both inclusion and exclusion criteria.
Pre-pregnant women; pre-pregnant women; adolescents; maternal and child health care service providers

20 Intervention(s), exposure(s)
Give full and clear descriptions of the nature of the interventions or the exposures to be reviewed
Interventions that linked care from pre-pregnancy to pregnancy

21 Comparator(s)/control
Where relevant, give details of the alternatives against which the main subject/topic of the review will be compared (e.g. another intervention or a non-exposed control group).
Interventions that did not link care from pre-pregnancy to pregnancy

22 Types of study to be included initially
Give details of the study designs to be included in the review. If there are no restrictions on the types of study design eligible for inclusion, this should be stated.

We will include randomised controlled trials and quasi-randomized trials conducted in low- and middle- income countries for this study. We will also include observational studies where the study are missing.

23 **Context**
Give summary details of the setting and other relevant characteristics which help define the inclusion or exclusion criteria.
Continuum of care (CoC) has been a key to reducing maternal, newborn and child mortality risks. CoC is a combination of care in time dimension (from pre-pregnancy to motherhood/childhood) and space dimensions (from family/community care to clinical care). The World Health Organization stated that maternal, newborn and child health interventions could be effective only if they are carried out together. However, most of the studies in CoC have focused on pregnancy, birth, and postnatal periods of mother and child. Almost no attempt has been made to assess the maternal/child health impact of interventions that linked care from pre-pregnancy to pregnancy. We thus aim to conduct a systematic review to identify those interventions, and to examine the impact on maternal health outcomes or service utilization outcomes.

24 **Primary outcome(s)**
Give the most important outcomes.
- Maternal morbidity (anaemia, postpartum sepsis, STIs, neural tube defects) - Unmet need of pregnancy - Maternal service utilization - Maternal service coverage
Give information on timing and effect measures, as appropriate.

25 **Secondary outcomes**
List any additional outcomes that will be addressed. If there are no secondary outcomes enter None.
- Contraceptive use - Post abortion care - Routine immunization - Vitamin A supplementation - Elective abortion
Give information on timing and effect measures, as appropriate.

26 **Data extraction, (selection and coding)**
Give the procedure for selecting studies for the review and extracting data, including the number of researchers involved and how discrepancies will be resolved. List the data to be extracted.
We will prepare an inventory of the interventions aims to reduce maternal morbidity or unmet need of pregnancy using the key interventions recommended by RMNCH (RMNCH 2012). For literature search, we will use appropriate key words, accepted MeSH words, and combinations thereof. One search approach employed broad serach terms (e.g. ("Pre-pregnancy"[MeSH] OR adolescent OR mother), and is combined with serach terms specific for interventions, (e.g. ("Family planning" [MeSH] OR contraception OR spacing). To supplement the search, we will also review reports published by agencies including RMNCH, WHO, UNICEF and the World Bank. We will conduct a further snow-balling search through hand searching or references from identified studies.

27 **Risk of bias (quality) assessment**
State whether and how risk of bias will be assessed, how the quality of individual studies will be assessed, and whether and how this will influence the planned synthesis.
We will use Cochrane's "risk of bias" tool to assess the bias of RCTs. Assessment will be for seven domains: sequence generation, allocation concealment, blinding of participants and outcome assessors, incomplete outcome data, selective outcome reporting, and other potential threats to validity.

28 **Strategy for data synthesis**
Give the planned general approach to be used, for example whether the data to be used will be aggregate or at the level of individual participants, and whether a quantitative or narrative (descriptive) synthesis is planned. Where appropriate a brief outline of analytic approach should be given.
We will descriptively analyze the extracted data according to the types of intervention, study design, participants, country, outcome, and risk of bias. We will examine the risk ratio based on the nature of outcome variables reported. If we have enough RCTs with quality data reported, we will conduct meta analyses to find the effectiveness of intervention. In event of lacking of similar data on outcome variable, we will run a narrative summary.

29 **Analysis of subgroups or subsets**
Give any planned exploration of subgroups or subsets within the review. 'None planned' is a valid response if no subgroup analyses are planned.
None planned

Review general information

30 **Type of review**
Select the type of review from the drop down list.
Intervention

31 **Language**
Select the language(s) in which the review is being written and will be made available, from the drop down list. Use the control key to select more than one language.
English
Will a summary/abstract be made available in English?
Yes

32 **Country**
Select the country in which the review is being carried out from the drop down list. For multi-national collaborations select all the countries involved. Use the control key to select more than one country.
Japan

33 **Other registration details**
Give the name of any organisation where the systematic review title or protocol is registered together with any unique identification number assigned. If extracted data will be stored and made available through a repository such as the Systematic Review Data Repository (SRDR), details and a link should be included here.

34 **Reference and/or URL for published protocol**
Give the citation for the published protocol, if there is one.
Give the link to the published protocol, if there is one. This may be to an external site or to a protocol deposited with CRD in pdf format.

I give permission for this file to be made publicly available
Yes

35 **Dissemination plans**
Give brief details of plans for communicating essential messages from the review to the appropriate audiences.
Do you intend to publish the review on completion?
Yes

36 **Keywords**
Give words or phrases that best describe the review. (One word per box, create a new box for each term)
continuum of care
pre-pregnancy care
adolescents
reproductive health
antenatal care

37 **Details of any existing review of the same topic by the same authors**
Give details of earlier versions of the systematic review if an update of an existing review is being registered, including full bibliographic reference if possible.
No earlier version

38 **Current review status**
Review status should be updated when the review is completed and when it is published.
Ongoing

39 **Any additional information**
Provide any further information the review team consider relevant to the registration of the review.

40 **Details of final report/publication(s)**
This field should be left empty until details of the completed review are available.
Give the full citation for the final report or publication of the systematic review.
Give the URL where available.

UNIVERSITY of Yor
Centre for Reviews and
