# Supplementary material for: Effectiveness of Continuum of Care—Linking Pre-Pregnancy Care and Pregnancy Care to Improve Neonatal and Perinatal Mortality: A Systematic Review and Meta-Analysis
Source: PLoS One. 2016 Oct 27;11(10):e0164965. doi: 10.1371/journal.pone.0164965 (PMC5082954; doi:10.1371/journal.pone.0164965)
Supplement: S3 File — (DOCX) [file pone.0164965.s003.docx]

**Text S3**

**Effectiveness of Continuum of Care－Linking Pre-Pregnancy Care and Pregnancy Care to Improve Neonatal and Perinatal Mortality: A Systematic Review and Meta-Analysis**

**Research Question**

**Does linkages between pre-pregnancy and pregnancy care reduce maternal, neonatal, or perinatal mortality in low- and middle- income countries?**

***Strategy 1: Search terms for “participant”***

("mothers"[MeSH Terms]　OR "women"[MeSH Terms] OR "female"[MeSH Terms] OR "adolescent"[MeSH Terms] OR "pregnant women"[MeSH Terms])

***Strategy 2: Search terms for “intervention”***

("family planning services"[MeSH Terms] OR "condoms"[MeSH Terms] OR “diaphragm”[MeSH Terms] OR "contraceptives, oral"[MeSH Terms] OR "contraceptives, postcoital"[MeSH Terms] OR (hormonal[All Fields] AND "injections"[MeSH Terms]) OR (long[All Fields] AND acting[All Fields] AND reversible[All Fields] AND "contraceptive agents"[MeSH Terms])OR("sexually transmitted diseases"[MeSH Terms] OR "syphilis"[MeSH Terms] OR "gonorrhea"[MeSH Terms])OR("folic acid"[MeSH Terms])OR("abortion, induced"[MeSH Terms] AND "counseling"[MeSH Terms])OR(post [title/abstract] AND ("abortion, induced"[MeSH Terms]) AND care[All Fields]))

***Strategy 3: Search terms for “Outcome”***

((“maternal mortality"[MeSH Terms] OR ("maternal"[All Fields] AND "mortality"[All Fields]) OR "maternal mortality"[All Fields])OR("perinatal mortality"[MeSH Terms] OR ("perinatal"[All Fields] AND "mortality"[All Fields]) OR "perinatal mortality"[All Fields])OR(("neonatal"[All Fields] AND "mortality"[All Fields]) OR "neonatal mortality"[All Fields]))OR (("maternal death"[MeSH Terms] OR ("maternal"[All Fields] AND "death"[All Fields]) OR "maternal death"[All Fields])OR("perinatal death"[MeSH Terms] OR ("perinatal"[All Fields] AND "death"[All Fields]) OR "perinatal death"[All Fields])OR(("neonatal"[All Fields] AND "death"[All Fields]) OR "neonatal death"[All Fields]))

***Strategy 4: Search terms for “Study design”***

("randomized controlled trials as topic"[MeSH Terms] OR "random allocation"[MeSH Terms])
